# Hydro-Electric Baths; Ionic Medication

**Published:** 1913-05-31

**Authors:** Alfred C. Norman

**Affiliations:** House Surgeon at Durham County Eye Infirmary.


### ELECTRICITY IN MODERN MEDICINE.*

### XXX.—Hydro-Electric Baths; Ionic Medication.

By ALFRED C. NORMAN, M.D. Edin., House Surgeon at Durham County Eye Infirmary.

The hydro-electric bath is a convenient method of applying galvanic, faradic, or sinusoidal currents to larger areas of the body than can be done satisfactorily by means of ordinary pad electrodes. Tap water makes an ideal electrode. By virtue of the salts it contains in solution it is a fairly good conductor of electricity; it adapts itself perfectly to any part of the body immersed in it; and it keeps the skin warm and well moistened during the whole time the current is applied. Hydro-electric baths may be either dipolar or monopolar.

### The Dipolar Bath.

When two electrodes are immersed in one bath it is known as a dipolar bath, one electrode being connected with the positive the other with the negative pole of the source of current. This method is used chiefly in the application of electricity to the whole body, the patient (with the exception of his head) being immersed in a large porcelain bath having an electrode at each end. (A small dipolar bath is sometimes used in the treatment of a single limb, as, for instance, the forearm in lead paralysis, but we shall see later that monopolar baths are more scientific and exact for this purpose.)

In giving an electric bath the following precautions must be observed. The water should be kept at a temperature of about 100° F. The patient's head and feet should not be in contact with the metal electrodes. Shocks should be carefully guarded against, for, the patient's wet skin being a good conductor and his whole body being exposed to the current, a shock might easily prove fatal. The current must never be switched on or off at full strength, but must be gradually increased or decreased by means of a finely graduated rheostat, and, above all, it must be reduced to zero before being reversed, for we have seen that a sudden make or break or reversal produces a maximum of physiological effect. For this reason connecting cords must be sound and must be firmly attached to the electrodes and terminals respectively.

A milliampere-meter must always be used when giving an electric bath, and it is a disadvantage of the dipolar bath that we have no means of knowing the proportion of current passing through the patient to that passing through the water, for the milliampere-meter reading only indicates the total current flowing. The more water there is in the bath the more current will it carry and consequently the less will pass through the patient. Obviously, it would not do to use a metal bath (unless very perfectly enamelled), for the current would all travel from electrode to electrode through the bath and the patient would receive none of it. If certain salts are dissolved in the water for purposes of medication the conductivity of the water will be increased, consequently, it will be necessary to increase the current strength (as indicated by the milliampere-meter) if the patient is to receive as much current as before. From all this it is obvious that water constitutes a good electrode for dipolar baths because its resistance is approximately similar, volume for volume, to that of the human body.

*Sources of Electricity for Electric Baths.*—The galvanic current may be given from large primary batteries or from batteries of accumulators, and the faradic current from a specially designed faradic coil, but by far the best method and the cheapest in the long run if current from the main is available is to use an "earth free" motor generator such as the Pantostat. This furnishes a galvanic or a sinusoidal faradic current perfectly safe from the risks of earth shock and is fitted with a good milliampere-meter and finely graded rheostats.

*Shunt Rheostats* must never be used to give current from the main in an electric bath because of the great risk of a fatal earth shock. This might happen if a patient touched a water tap or a gas bracket, or even if someone turned on the water or opened the exhaust pipe, for a stream of water without any metallic connection whatever will conduct electricity to earth. Apart from earth shocks, a short circuit in the rheostat itself might cut out the resistance in the main circuit, and so be the means of passing the full voltage from the mains through the patient. This is most likely to happen when an incandescent lamp is used as the main resistance, for a broken lamp filament may fall in such a way as to cut out its own resistance and yet restore the circuit. By using four lamps of suitable resistance in series this danger might be minimised, but still the method is better left alone.

### Monopolar Baths.

In the monopolar method each bath contains but one electrode, consequently, at least two baths are required—one negative, the other positive—and the patient places a limb in each bath. Small earthenware baths are made for the purpose and are shaped to take an arm and a leg respectively. If a patient places, say, his arm in one bath and his leg in another all the current flowing in the circuit must pass through his body, consequently, the milliampere-meter will indicate the exact amount of current he is getting.

*Schnee's Four-Cell Bath Outfit* is a very complete set of apparatus for utilising the monopolar method.

* Previous articles appeared on Nov. 11, 25, Dec. 9, 30, 1911; Jan. 13, 27, Feb. 17, March 9, 30, April 20, May 4, 25, June 8, July 6, Aug. 3, 17, 31, Sept. 28, Oct. 12, 26, Nov. 9, 30, Dec. 14, 1912; Jan. 11, Feb. 1, March 1, April 12, and May 3, 17.

It consists of two arm baths, two leg baths, a special chair, a motor generator with milli-ampere-meter and rheostats (the Pantostat may be used), and a special switchboard. The patient sits on the chair with his arms and legs each in a separate bath, the motor generator furnishes a galvanic or a faradic (sinusoidal) current as may be required, and the special switchboard can be used to make each bath positive or negative at will.

By means of the Schnee baths it is possible to concentrate the current in almost any part of the body—a special set of diagrams being supplied as a guide in this respect. For instance, if the patient places his left arm and his legs in separate negative baths and his right arm in a positive bath, the current density will be greatest in his right arm. The possible combinations are almost endless and the method is particularly useful in the treatment of paralysis of the extremities.

In all forms of electric-bath treatment the patient experiences a stinging sensation at the line represented by the upper level of the water. Tousey of New York has pointed out that this sensation can be obviated by surrounding the parts with a woollen garment—socks, for instance, when leg baths are to be given.

### IONIC MEDICATION.

Ionic medication consists in utilising the electrolytic action of continuous currents to convey various chemicals into the tissues of the human body. The advantages of this method over the ordinary method of medication by the mouth are very considerable when a local action of a drug that can be ionised is desired. We can obtain a greater local concentration of the drug than could be obtained *via* the blood stream, and the action is more lasting because the drug is actually carried into the individual cells of the body instead of remaining in the lymph spaces and capillaries.

*Sources of Electricity.*—A continuous current must be used. If an alternating current were employed the electrolytic result would be *nil*, for every impulse in one direction would be promptly neutralised by the action of the succeeding impulse in an opposite direction. A battery of (at least thirty) dry or wet cells may be used, or a shunt rheostat and current from the mains, or a motor transformer such as the Pantostat. It is essential that a milliampere-meter be employed and the current must be carefully regulated by means of a suitable rheostat.

*Theoretical Considerations.*—When an electric current is passed through a solution of a salt the latter is dissociated into its chemical components, and these, being electrically charged, are termed "ions." Now, certain ions are driven away from the positive pole only, and certain other ions from the negative pole only; consequently, when we wish to drive any kind of ion into the human body, we must employ the right pole.

*Practical Applications.*—For localised applications the best active electrode is a pad of cotton wool well moistened with a solution of the chemical to be introduced and backed by a piece of metal or carbon, and the neutral electrode may conveniently be an arm or a leg bath. If metal is used to back the electrode it must be the same as the salt in solution; failing this, carbon must be employed, as it is not acted upon by the chemical solution. When it is desired to treat extensive areas, as, for instance, the introduction of lithium into a gouty extremity, the limb is immersed in a solution of lithium sulphate in a monopolar bath; an electrode connected with the positive pole of the battery is suspended in the fluid, this forming the active electrode. The other pole of the battery is connected with a bath containing a weak solution of sodium bicarbonate, thus forming the neutral electrode; the patient places a limb in this bath to complete the circuit.

The beginner must remember that all metals and alkaloids are driven into the tissues from the positive electrode and all acid radicals from the negative electrode, and he must be careful to connect what he intends to be his active electrode to the proper pole of the battery. The following is a list of ions that are often used and the poles from which they are driven:—

| Ions from Positive Pole. | Ions from Negative Pole. |
|---|---|
| Zinc. | Chlorine. |
| Lithium. | Salicylic. |
| Copper. | Iodine. |
| Magnesium. | |
| Cocaine. | |
| Quinine. | |

*Salts usually employed.*—To introduce the ions of zinc, lithium, copper, or magnesium we should take a 1 per cent. or a 2 per cent. solution of the sulphate of the desired metal and apply it through the positive electrode. Solutions of quinine sulphate or cocaine hydrochloride may be employed in a similar manner. To introduce chlorine, salicylic, or iodine ions the sodium salt in 1 or 2 per cent. solution should be employed at the negative electrode.

*Strength of Current.*—One milliampere applied over an area of one square centimetre will have precisely the same effect on the tissues beneath the electrode as twenty milliamperes over twenty square centimetres. It follows, therefore, that in treating large areas we must use strong currents and *vice versa.* Most patients will not stand, without an anæsthetic, a current of greater density than two milliamperes per square centimetre, and this is the strength usually employed in practice.

There is no question that ionic medication is of great value in treating a considerable number of diseases, and it has been proved experimentally that the ions are actually driven a considerable distance into the tissues. For instance, ferricyanide ions have been introduced into the knee joint and have been demonstrated even in the cartilages.

Zinc ions for rodent ulcer; salicylic ions for sciatica, neuralgia, and arthritis; magnesium ions for warts; and chlorine ions for softening cicatricial tissue have now a definite and assured place in modern therapeutics, and it is only a question of time before many others are added to the list.

*(To be concluded.)*

